# Return-to-Play Timeline and Recovery Predictors After COVID-19 Infection in Elite Football Players

**DOI:** 10.3390/sports13050147

**Published:** 2025-05-15

**Authors:** Agnes Sziva, Zsuzsanna Kives, Zsolt Szelid

**Affiliations:** 1 Doctoral School of Health Sciences, Faculty of Health Sciences, University of Pécs, 7622 Pécs, Hungary; sziva.agnes@gmail.com; 2 Institute for Health Insurance, Faculty of Health Sciences, University of Pécs, 7622 Pécs, Hungary; zsuzsa.kives@etk.pte.hu; 3 Hungarian Football Association, 1112 Budapest, Hungary; 4 Department of Sports Medicine and Digital Health, Faculty of Health and Sports Sciences, University of Győr, 9024 Győr, Hungary

**Keywords:** elite football, COVID-19, return-to-play, performance recovery, SARS-CoV-2 infection

## Abstract

The pandemic period significantly impacted professional football, leading to mandatory SARS-CoV-2 testing and quarantine. Our study aimed to examine the factors influencing time of recovery after a positive test, including return-to-training (RTT) and return-to-first-match (RTFM) of male football players in a first-division Hungarian team between 8 May 2020 and 30 June 2022. Infection was determined using mandatory RT-PCR testing 3 times per week, which later decreased to 1 to 2 times per week, in 55 elite players. A self-administered questionnaire was utilized based on the U.S. Department of Health and Human Services symptom list and modified with relevant factors of return-to-play in football. The incidence of SARS-CoV-2-positive players in the three consecutive years was 5.26; 21.43 and 45.71%. Mild symptoms were present in test-positive players, completing the questionnaire (*n* = 31), predominantly loss of smell and dry cough. Post-infection fatigue levels correlated with the perceived performance decline. In players with precisely documented dates (*n* = 18), the average RTT was 18.7 days, while the RTFM was 67.3 days. Older players returned to training faster than their younger counterparts and the RT-PCR Ct number had a weak negative correlation with RTFM. Mental support was provided by family and friends in 68% of the players. This study highlights the variability in return-to-play timelines and the role of age, symptom severity and mental help in recovery and emphasizes the need for individualized rehabilitation in elite football.

## 1. Introduction

Professional sports, including football, underwent significant transformations during the COVID-19 pandemic. This unfortunate period gave a unique opportunity for healthcare professionals to examine the physical and mental aspects of mandatory rest and if viral infection was present, the return-to-play of numerous professional athletes. In Hungary, all organized football trainings and matches were suspended between 15 March 2020 and 8 May 2020 [1] to prevent spread of the virus and fatal infection-related cardiovascular complications [2]. In this period, all players were assigned exclusively home-based training programs to prevent detraining-related body composition changes, and reduced performance, which was previously described in football players following prolonged rest [3]. From May 2020, first-division team training and championship matches resumed under strict preventive measures, including regular SARS-CoV-2 PCR (polymerase chain reaction) testing of players and staff based on nasopharyngeal samples, enabling early detection of the disease. According to the regulations of the Hungarian Football Association (HFA) during the first year of the pandemic (2020), PCR testing in the Hungarian first division was mandatory two times a week, and then in the subsequent period (in 2021 to June 2022), testing was carried out prior to matches, once or twice weekly [1]. Additional PCR tests and preventive measures were, however, introduced in several clubs. Home quarantine was mandatory for all athletes during the lockdown, while in the latter pandemic period, this was only for test-positive players.

Return-to-play of previously COVID-19-positive athletes in Hungary was regulated at several levels. National prevention standards for all registered athletes were issued by the National Institute for Sports Medicine (NISM), which represents the national authority for public health in sport. The NISM mainly focused on isolation measures and minimal requirements of return-to-play (RTP) medical examinations, including resting 12-lead ECG, blood pressure measurement and blood tests for troponin level and signs of infection [4]. National prevention measures for soccer players in Hungary were regulated by the Hungarian Football Association, whose guidelines were in line with the actual medical regulations, issued by the Union of European Football Associations (UEFA). The UEFA regulations were, however, only mandatory prior to UEFA matches and not in the national championships, while regulations, implemented by the HFA, were mandatory for all players participating in the Hungarian first-division football championship. In addition to the PCR testing schedule mandated by the HFA (PCR tests twice a week for screening), some clubs supplemented this protocol with additional weekly PCR tests. This allowed for the precise tracking of players’ health status and enabled the measurement of return-to-training and return-to-match timelines with exceptional accuracy. Therefore, regulations in Hungary were in line, but stricter, than those suggested by the UEFA, which made it possible to organize the UEFA Europe League final in Budapest with a full stadium of spectators [5], while this was still forbidden in other stadiums in Europe. COVID-19-positive athletes, irrespective to the symptoms, underwent complete rest, which can result in significant muscle mass loss within just a few days, followed by cardiovascular and pulmonary endurance decline few weeks later [6]. Similarly, training cessation during the lockdown was shown to reduce physical performance, irrespective to the playing position and the COVID-19 status of the players [7]. In the general population, COVID-19 patients suffered from a wide range of symptoms, including coughing, respiratory symptoms, loss of smell and taste, muscle pain, elevated body temperature, and fever [8]. In athletes with acute COVID-19 infection, the most common symptoms were fatigue, headache and cough according to a large UK database [9], and the majority of athletes, including soccer players, presented only mild or no symptoms, with no need for hospitalization [10,11]. In a large self-reported, app-based study, early disease features were predictive of the duration of long COVID-19-related symptoms [12] and prolonged return-to-sport was described in athletes with more severe and a greater range of symptoms during both the acute and the post-infection phase [13]. In athletes, including soccer players, fatigue was described as the most predominant symptom, which hindered return-to-play and full performance capacity regain after the infection [9,11,14,15]. In our study, a Hungarian first-division football team was examined, which was involved in the UEFA Europa League in this period. Therefore, both the rigorous regular national testing regime and the UEFA pre-match and RTP pandemic regulations were mandatory, which were extended by additional COVID-19 testing according to the local club regulation. We aimed to determine the time scales required to return-to-sport following infection in elite football players following COVID-19 infection including the timeline for reintegrating into official training sessions, and regaining performance capacity to play their first match. Although international and national RTP regulations did not include sport-specific tests upon return-to-play, local regulation, implemented in our examined football team, included some sport-specific examinations before return-to-training, like cardiopulmonary exercise tests and a graduated return-to-play protocol, and was published at the early stage of the pandemics [16]. This infographic-based protocol relied on objective parameters, including percentage of maximal heart rate, training volume and rated perceived exertion. The aim of this study was also to explore different factors influencing these time periods, with a particular focus on age, symptom severity, and perceived mental support during recovery. Regular virus testing also provided data on the incidence of infection rates among athletes of a first-division Hungarian football club during the pandemic years.

## 2. Materials and Methods

### 2.1. Participants

The study included all adult male football players (aged 18 years and above) who were officially registered with a first-division Hungarian football club, which was included in this paper, between 8 May 2020 and 30 June 2022. Participation in the SARS-CoV-2 PCR testing was part of the club’s health protocol and it was mandatory for all players. No formal exclusion criteria were applied for this dataset. Throughout the complete study period, a total of 55 different players were affiliated with the club and participated in the mandatory testing protocol. However, due to player transfers, loan agreements, and expiring contracts, the squad composition changed across different years. The numbers of players in the different periods were as follows: in May–Dec. 2020, *n* = 38 players, in Jan.–Dec. 2021, *n* = 42 players and in Jan.–Jun. 2022, *n* = 35 players. Questionnaires were distributed retrospectively to players who had been members of the club between May 2020 and June 2022 and were tested at least once positive for SARS-CoV-2 during this period.

### 2.2. Study Design and Data Collection

We conducted a quantitative, descriptive follow-up study in a Hungarian first-division football team. Nasopharyngeal swab collection for SARS-CoV-2 testing was consistently performed by the same trained medical personnel throughout the entire pandemic period and SARS-CoV-2 RT-PCR was performed in the laboratory of Synlab Hungary Ltd. Pooled RT-PCR tests and rapid antigen test results were not accepted. A SARS-CoV-2 RT-PCR test was considered positive with a cycle threshold (Ct) < 38. The twice-weekly testing was mandatory by the HFA and the examined club supplemented the weekly testing with one additional testing session per week for all players between May and Dec. 2020. Therefore, the number of SARS-CoV-2 PCR tests via nasopharyngeal swab was 3 times per week in each player in the examined club. Later, the HFA regulation requested mandatory twice-weekly testing until May 2021. Following this, the HFA guideline requested only pre-match testing. This club, however, continued the testing three times per week until May 2021, which then turned to regular testing two times per week for all players. Therefore, in the period of Jan.–Dec. 2021, tests 2–3 times per week were performed in the examined players. In the later period, in Jan.–Jun. 2022, the club changed to pre-match testing, which included testing each player 1–2 times per week. Players with positive test results immediately started mandatory home quarantine and returned only under medical guidelines approved by the Hungarian Football Association, requiring two consecutive negative SARS-CoV-2 PCR tests at least 48 h apart. Based on these protocols, three key time intervals were identified during return-to-play in our study:

Return-to-training (RTT): The number of days between the first positive SARS-CoV-2 PCR test in the current infection period and the first training session.

Return-to-first-match (RTFM): The number of days between the first positive PCR test in the current infection period and the first match following quarantine.

Return-to-first-match from return-to-training (RTFM-T): The number of days between the return-to-training date and the first match date.

To evaluate the symptoms of football players who tested positive at least once during the examined period, a self-administered questionnaire was distributed, which was based on a symptom checklist provided by the U.S. Department of Health and Human Services [17] and adapted to football players and return-to-sport variables. The first section of the questionnaire focused on peak symptoms during the acute phase of the infection and the mental aspects of quarantine, while the second section addressed symptoms emerging during the recovery period and included players’ perceived evaluations of their health and physical performance. Post-COVID-19 questions targeted long-term COVID-19 symptoms, including the physical and mental impacts of the disease, and its influence on players’ sports careers. Fatigue levels following return-to-sport were rated by players on a Likert scale from 1 (not fatigued at all) to 5 (very fatigued). The physical performance impact of COVID-19 was similarly rated on a Likert scale from 1 (no impact) to 5 (significant performance decline). Player position was also determined using 4 standard categories: forward, midfielder, defender and goalkeeper. The questionnaire was available in both online and paper formats and was provided in Hungarian and English to include foreign players. Participation in the study was voluntary and conducted with the approval of the football club. Player consent was a prerequisite for study participation. The research was approved by the Scientific and Research Ethics Committee of the Hungarian Medical Research Council (TUKEB), under registration number BM/17634-3/2023.

Symptom comparison and analysis of the timeframes for return-to-sport were conducted only for players with an exactly identifiable SARS-CoV-2 infection date confirmed by RT-PCR testing, and who completed the questionnaire in a fully evaluable manner. Symptom evaluation was based on the players’ first recorded infection date. The duration of both return-to-training and return-to-first-match was calculated using the date of the first confirmed COVID-19 infection in the actual infection period.

### 2.3. Statistical Analysis

Data were recorded and organized using Microsoft Excel (Microsoft Corporation, version 2402, build 16.0.17328.20124, 64-bit), which was also used for basic mathematical operations. All statistical analyses were performed using SPSS version 28.0 for Windows (IBM Corp., Armonk, NY, USA). The distribution of variables was assessed using the Shapiro–Wilk test, which indicated that the data were not normally distributed. Therefore, non-parametric statistical methods were applied. Descriptive statistics are reported as mean ± standard deviation, median, minimum–maximum range, and frequency. Associations between continuous or ordinal variables were analyzed using Spearman’s rank correlation coefficient (ρ). Statistical tests were two-sided, and significance was set at *p* < 0.05. The effect sizes were classified by correlation-based measures (Spearman’s ρ), with the following thresholds: small effect: r = 0.10–0.29 (or −0.10 to −0.29), medium effect: r = 0.30–0.49 (or −0.30 to −0.49) and large effect: r ≥ 0.50 (or r ≤ −0.50).

## 3. Results

In the pandemic period, the total number of tested football players was 55. However, the exact number of tested players varied in specific years (see Chapter 2.1). The incidence of players with positive test results is presented in Table 1, which also shows the cumulative number of tests and positive test results in certain pandemic years in a specific first-division football team. Using the described testing regimes, the incidence rates of virus-positive players clearly increased during the pandemic years (Table 1), from 5.26 to 45.71%. The incidence rate of SARS-CoV-2-positive RT-PCR tests among players also increased in successive years 2020, 2021 and 2022, starting with 0.62% and ending with 4.39% in the final pandemic year.

Among the 55 regularly tested players, 31 players had at least one positive SARS-CoV-2 RT-PCR test result. The median RT-PCR Ct value of all positive tests was 24.88. All players who tested positive for SARS-CoV-2 at least once during the examined period completed the self-reported questionnaire (*n* = 31). The average age of these players was 27.2 ± 4.4 years, ranging between 18 and 34 years. Among the SARS-CoV-2-positive players, 19 players tested positive once, 9 players twice, 2 players three times, and 1 player four times during the 112 weeks of the study period. A weak negative correlation was observed between the players’ age and the number of infections during the pandemic period (r = −0.364; *p* = 0.044), suggesting that the frequency of reinfections decreased with age. During the quarantine period, the most commonly reported symptoms were loss of taste, coughing, and fever or elevated body temperature (Table 2). The number of symptoms varied among players: 3 players reported no symptoms, 2 players reported only one symptom, 11 players reported two symptoms, 6 players reported three symptoms, 5 players reported four symptoms, and 4 players reported five or more symptoms. The median number of symptoms during the infection period was two. No significant correlations were found between the number of symptoms and age (r = −0.063; *p* = 0.738) or the number of infections (r = 0.208; *p* = 0.262).

During quarantine, 20 players reported no mental difficulties, 9 players experienced impatience, 9 expressed anxiety, and 2 exhibited heightened symptom-monitoring behaviors. In the post-COVID-19 period, the players rated their fatigue levels on a 1 to 5 Likert scale, with an average score of 2.45 (median: 2, minimum: 1, maximum: 4). Fatigue levels showed no correlation with the number of symptoms experienced during the acute infection period (r = −0.125; *p* = 0.501) or age (r = −0.063; *p* = 0.738). Upon returning to sport, most players (18 individuals) reported no residual symptoms. Among those who experienced symptoms, five players cited muscle pain, six reported fatigue, one mentioned sleep disturbances, two experienced loss of smell, and two noted cognitive fog. Six months post-infection, six players continued to experience symptoms, including chest pain (two players), coughing (two players), shortness of breath (one player), and abdominal cramps (one player).

The players assessed the impact of COVID-19 on physical performance on a 5-point scale, where 1 indicated “no impact” and 5 indicated “significant performance decline”. The average score was 1.8 ± 0.98 (median: 2, minimum: 1, maximum: 5). Fifteen players reported no impact on their performance, while one player experienced significant decline. Performance decline perception showed no correlation with age (r = −0.243; *p* = 0.188) or symptom count (r = 0.152; *p* = 0.414). However, there was a moderate positive correlation between fatigue levels and perceived performance decline (r = 0.655; *p* < 0.001), indicating that higher post-COVID-19 fatigue levels were associated with a greater perceived decline in performance. Regarding recovery time, 30 players reported regaining their pre-infection performance levels within one month, while one player felt fully recovered only after 3–4 months. When asked if mental support could have mitigated the performance decline, 14 players believed it would have had no effect, 12 players found it potentially helpful, and 5 were uncertain. Twenty-one players (68%) received mental support from their families and friends, while less players received help from the team (Table 3). Nine players reported receiving no support at all, eight relied solely on family and teammates and one player received only professional support, while thirteen players benefited from both family and professional mental support.

Based on the questionnaire responses, 22 players reported that the infection had no impact on their later sports career, while 9 players indicated it temporarily hindered their progress. Due to the small sample size, further analysis of this variable was not feasible.

The exact date of infection was identified in 18 players, enabling a comparison of symptoms and return-to-sport timelines within this subgroup. Following the first infection, the average duration from the first training session to the first match was 48.7 days, with considerable variation. For the second infection, which affected only four players, the average duration from the first training session to the first match increased to 64.2 days (Table 4).

The time between the first positive test and the return-to-training showed a moderate negative correlation with age (r = −0.575; *p* = 0.013), indicating that older players returned to training more quickly. The severity of SARS-CoV-2 RT-PCR test positivity, confirmed by the Ct value, showed a weak negative correlation (r = −0,377; *p* = 0,0319) with RTFM, but no correlation was observed with RTT times. This indicates that the severity of the test result in positive players was not associated with the time of return-to-training, but it slightly correlated with the return-to-first-match. The duration until return also demonstrated a moderate positive correlation with the number of symptoms experienced, suggesting that players with more symptoms during the acute phase required longer recovery periods before resuming training (r = 0.487; *p* = 0.040). For the time interval between the first training session and the first match, two outliers (135 days and 225 days) were excluded, leaving data from 16 players for analysis. The adjusted dataset revealed no significant correlation between the time from training resumption to the first match and either age (r = 0.335; *p* = 0.240) or the number of symptoms (r = −0.413; *p* = 0.112). RTFP times were also determined in different player positions: in midfielders (*n* = 6), 40.5 ± 25.6 days; in forwards (*n* = 7), 47.8 ± 36.0 days; in defenders (*n* = 6), 23.2 ± 8.5 days; in goalkeepers (*n* = 3), 133.3 ± 99.9 days. However, the association between a player’s position and return times was not analyzed in this paper because of the relatively low number of players in the different position subgroups.

## 4. Discussion

The COVID-19 pandemic had an important impact on elite sport life, including the mandatory rest period for all athletes during lockdown and later strict virus testing regimes with mandatory rest and controlled return-to-play for COVID-19-positive players to prevent fatal complications [2]. The strict preventive measures introduced in European football resulted in low virus transmission risk during matches [18] and even in athletes with severe symptoms, virus-related myocardial damage risk was low [19]. In this descriptive follow-up study, professional soccer players in a Hungarian first-division Hungarian club were included and self-administered questionnaires were utilized to reveal the factors that influenced the return-to-sport duration following infection. In the examined club, SARS-CoV-2 RT-PCR test results were obtained for all first-division players during the pandemic period, with mandatory nasopharyngeal swab testing for RT-PCR three times a week in 2020, and with mandatory pre-match testing 1–2 times a week in the following years until 30 June 2022. Incidence of COVID-19-positive players increased during the pandemic years, from 5.26% to 45.71%, which is much higher than the reported incidence in professional football players in studies from Greece and Danmark [20,21]. The incidence rate of SARS-CoV-2-positive RT-PCR tests of all players also increased from 0.62 to 4.39% in the years following 2020. In our study, the ratio of players to number of SARS-CoV-2 tests was 1:83, while in a Danish study, it was 1:8.7. Therefore, the higher incidences reported in our study compared to the earlier published study with a Danish professional football cohort [21] may be explained by the ten times more frequent testing in our study, resulting in a greater number of positive test results from the players with prolonged positivity. In the questionnaires utilized in our study, identification of acute and post-COVID-19 symptoms was based on the U.S. Department of Health and Human Services list [22], modified in accordance with the factors influencing return-to-play in football. Previous studies in non-athletic populations found diverse post-COVID-19 symptoms [23], while in athletes, the main symptoms during the acute phase were fatigue, dry cough and headache [9]. The most frequent symptoms we detected during the acute infection were loss of smell and dry cough. Additionally, many players experienced fever or elevated body temperature, loss of taste, muscle pain, and sore throat. The soccer players included in our study exhibited mild symptoms during the acute phase of the disease, similar to earlier observations in elite athletes [9,10,14,15], and 9,6% of players were asymptomatic, which is also similar to the previous observations in athletes who tested positive [13]. Sudre et al. (2021) previously confirmed that the presence and number of certain acute symptoms, including fatigue, headache, dyspnea, hoarse voice and myalgia, are predictive of prolonged symptoms after the acute infection and long-lasting symptoms were also associated with the age and sex of the patient [12]. The majority of players in our study remained asymptomatic when resuming training. Those who experienced symptoms upon return-to-sport predominantly reported fatigue and muscle pain. During the pandemic period, mandatory rest was prescribed for test-positive players, which itself was previously shown to result in several negative consequences in soccer, including reduced performance, decreased maximal oxygen consumption, decreased time to exhaustion, impaired running performance, worsening in football-specific yo-yo intermittent recovery test results [3,24] and decreased cardiac output, and stroke volume [25,26,27]. The moratorium on team training sessions during lockdown decreased aerobic abilities upon return-to-sport in symptomless elite handball [28] and in adolescent soccer players [29]. However, in professional adult soccer players, the results of previous studies on the effect of detraining on return-to-sport were inconclusive. Athletes who underwent 4-week rest periods from training exhibited similar endurance performance and even greater muscle strength compared to pre-rest levels [30], and when compared to the usual offseason 5-week breaks, even a 7-week mandatory detraining period was shown to result in better cardiorespiratory fitness levels and lower limb muscle strength in these athletes [31]. A longer, 9-week mandatory quarantine duration was reported to worsen physical performance compared to the offseason breaks [32]. Therefore, these previous studies on adult professional soccer players suggest that mandatory rest may differ from the usual offseason breaks in terms of certain physical fitness outcomes upon return-to-sport, which may be partially explained by the regular, high-intensity home-based exercises during mandatory rest periods, sometimes in contrast to medical advice. However, during the lockdown in the pandemic period, large cohort studies on professional football players (20 teams in LaLiga) showed that lack of team training for a similarly longer period (60 days) led to a decline in the majority of the performance variables when returning to play [33], which was shown to be partially position-dependent [7] and influenced by several player-specific factors [34]. Home-based physical training during mandatory rest has an impact not only on physical performance, but also may prevent mental health decline, as shown in young soccer players [35]. Mental conditions were important factors during the pandemic period and were influenced by several factors, including home advantage, absence of dominant players from the team, changes in substitution rules in matches [36], changes in stadium rules and lack of spectators during the pandemic period [37]. The return-to-play for COVID-19-positive players in Europe was regulated by the actual UEFA return-to-play guidelines [38], which governed the players’ asymptomatic return-to-matches based on the time elapsed after a positive SARS-CoV-2 RT-PCR test, ensuring player safety and preventing the spread of the virus to teammates and opponents [39]. In Hungary, return-to-play was allowed after a minimum of 10 days of quarantine, followed by two consecutive negative nasopharyngeal SARS-CoV-2 PCR tests, with a minimum period of 48 h apart [1]. The recent AWARE VIII study [13] on return-to-play following acute infective disease in high-intensity endurance sports, like football, defined consecutive times within return-to-sport, including return-to-training (RTT) and return-to-full-performance (RTFP). RTFP was based on the perceived sensation of the player. To be less subjective, instead of RTFP in our study, we used the time between a positive test result and the first match played in the first-division championship (return-to-first-match, RTFM). In our study, football players following the first positive SARS-CoV-2 test returned to training with a median time of 17 days, while the RTFM time was 45 days. In symptomatic players (*n* = 28), the median RTT was 16.5 days and RTFM was 45 days. In asymptomatic players, the RTT duration was 12 days and RTFM duration was 13 days (*n* = 3). The return-to-training times reported here are similar to previous observations, while the time of return-to-first-match was shorter than the previously described time of return-to-full-performance [13]. This might be explained by the high pressure on players by themselves, the team and the staff to play. Among players who contracted COVID-19 a second time, the median RTT was 6 days, and the median RTFM was 18 days. Return-to-play may be influenced by several potential player-related internal and external factors, including age, number and severity of symptoms during the acute phase of infection, reinfection rate, perceived football performance, long-COVID-19 symptoms and support by others after infection. Previous studies reported ongoing symptoms 3 months after the initial phase of infection in 15% of the athletes [19] and endurance sport, including football, was associated with prolonged return-to-full-performance time. Full performance was also hindered by the number of different symptoms during the acute phase of infection, including fatigue, loss of appetite, nausea, fever, chills, chest pain and headache [13]. Sport-specific technical performance indicators also showed differences upon return-to-play in the Big Five European national football leagues, and players needed around three matches to regain pre-infection match technical performance levels, irrespective of age, but performance decline was attenuated in the highest ranked elite players [40]. Pass performance was, however, influenced by age during the post-COVID-19 period in the Bundesliga and Serie A players, as its decline was more pronounced among players over 30, compared to younger players [41]. Results in an Italian study also revealed that playing position in football requires different training programs following return-to-play to regain full performance following COVID-19 infection [42]. In our study, a moderate correlation was observed between post-infection fatigue levels and perceived performance decline. Nevertheless, the majority of our players returned to their pre-infection performance level within a month. Most players stated that the infection did not affect their sports careers, although a few experienced temporary setbacks. Players with a wider range of symptoms took longer to return to sports than those with fewer symptoms. A moderate, negative correlation was detected between age of the players with both return-to-training and return-to-first-match. This means that older players returned more quickly than younger teammates, which may be explained by the improved technical and tactical skills in older soccer players, which was previously described to represent a possible compensation mechanism against physical performance decline related to aging [43]. In our study, the reinfection of players during the pandemic period did not correlate with the number of acute symptoms. The frequency of reinfections was, however, higher among younger players in our study, suggesting that reinfection rates decrease with age. The questionnaire revealed that players received various forms of mental support following virus infection and during return-to-play. Most players received support from family members and friends, while only a few players received support from teammates or club staff. Altogether, 42% (13/31) of players benefited from both family and professional mental support, 26% (8/31) relied solely on family and teammates and 1 player received only professional support. Opinions regarding the role of mental support in aiding performance recovery were surprisingly variable in the examined team. Nearly half of the players believed that mental support would not have been helpful during the post-infection period, while others considered it beneficial. These findings may provide insight into the different factors influencing return-to-sport after virus infection during the pandemic, emphasizing individual variability and the potential importance of mental support.

This study has several limitations that could be acknowledged. This sample represents one first-division team and is therefore not representative of the whole elite soccer population, meaning that the findings are limited to this study’s cohort and cannot be generalized. The relatively small sample size restricted the types of statistical tests that could be applied, reducing the reliability of certain conclusions. The reliance on self-reported data through questionnaires introduces the possibility of recall bias. Certain limitations were noted with the scales used to evaluate fatigue and perceived performance decline, which may have affected the accuracy and interpretation of the results. Additionally, no structured pre-season psychological assessments were conducted among the players during the study period. A further limitation is the lack of objective physical measurements to substantiate performance decline. Although returning to playing can be a potential objective indicator of return-to-sport in football, it is influenced by various tactical and coaching decisions, which are influenced by the head coach’s preferences, the player’s position, the availability of other players in the same role and the number of injuries in the team. Therefore, participation in an athlete’s first match should not be considered a direct measure of physical performance but rather interpreted as part of a complex return-to-play process. Our study did not examine some potentially influencing factors on physical activity and performance, like quality of life and sleep, which were described to be altered during the pandemic lockdown [44]. Increased sleep duration at night or napping during the day was described to be effective at improving physical and cognitive performance in athletes [45]; therefore, the inclusion of standardized questionnaires for sleep and quality of life is recommended in future research in athletic performance and recovery of elite football players.

## 5. Conclusions

In this study, the mandatory and rigorous COVID-19 testing regime for players in the Hungarian first-division championship during the pandemic period allowed the precise tracking of players’ COVID-19 status and enabled us to measure return-to-training and return-to-match timelines with exceptional accuracy. In the examined male first-division Hungarian football team, incidence of SARS-CoV-2-positive players gradually increased in the three consecutive years, while all players presented mild symptoms. Post-infection fatigue levels correlated with perceived performance decline. The average time of return-to-training was 18.7 days and return-to-first-match was 67.3 days, while older players returned to training faster than their younger counterparts. The intensity of virus positivity weakly correlated with the time of return-to-first-match. Mental support was mainly provided by family and friends. Our study, including the variability in return-to-play timelines and its association with personal and disease-related factors, including age, symptoms during and after the infection, underlines the importance of individualized rehabilitation strategies in elite football players.

## Figures and Tables

**Table 1:** Infection rates in the football team during the pandemic.

Time Periods	May–Dec. 2020	Jan–Dec. 2021	Jan.–Jun. 2022
Number of tested players	38	42	35
Number of RT-PCR tests	1458	2243	865
Number of positive RT-PCR tests	9	27	38
Number of positive players	2	9	16
Incidence of positive players (%)	5.26	21.43	45.71
Incidence of positive RT-PCR tests (%)	0.62	1.20	4.39

**Table 2:** Symptoms during the acute phase of COVID-19 infection (*n* = 31).

Symptoms	n	%
Loss of smell	17	54.8
Dry cough	16	51.6
Fever/hot flushes	13	41.9
Loss of taste	12	38.7
Muscle pain	12	38.7
Sore throat	11	35.5
Dizziness	1	3.2
Diarrhea	1	3.2
Nausea/vomiting	0	0
Palpitations	0	0
None	3	9.7

**Table 3:** Human resources of mental support for players (*n* = 31), where multiple responses were possible.

Source of Support	Players (n)
Family and friends	21
Teammates	8
Team doctor	10
Health staff	8
Coaching staff	8
Mental trainer	6
Other doctors	2

**Table 4:** Time from the positive test to return-to-training (RTT), return-to-first-match (RTFM), and return-to-first-match after training (RTFM-T) expressed in days.

Infection Sequence	Average	SD	Median	Range	IQR
First Infection (*n* = 18)					
RTT	18.7	8.8	17	2–42	13–23
RTFM	67.3	60.9	45	12–240	45–112
RTFM-T	48.7	59.9	26	1–225	7.5–88
Second Infection (*n* = 4)					
RTT	7.4	6.5	6	3–18	3–13.5
RTFM	72.5	73.3	18	6–159	9–146
RTFM-T	64.2	72.3	50	3–155	4.5–139

## Data Availability

The data that support the findings of this study are available from the corresponding author upon reasonable request. The data are not publicly available due to privacy or ethical restrictions.
